# A practical prognostic peripheral blood-based risk model for the evaluation of the likelihood of a response and survival of metastatic cancer patients treated with immune checkpoint inhibitors

**DOI:** 10.1186/s12885-023-11699-0

**Published:** 2023-12-04

**Authors:** Satu Tiainen, Veera Nurmela, Tuomas Selander, Patrik Turunen, Sanna Pasonen-Seppänen, Tiia Kettunen, Outi Kuittinen, Päivi Auvinen, Aino Rönkä

**Affiliations:** 1 Cancer Center, Kuopio University Hospital, Northern Savonia Healthcare Municipality, Kuopio, Finland; 2 Institute of Clinical Medicine, University of Eastern Finland, Kuopio, Finland; 3 Science Service Center, Kuopio University Hospital, Kuopio, Finland; 4 Institute of Biomedicine, University of Eastern Finland, Kuopio, Finland

**Keywords:** Immune checkpoint inhibitor, ICI, Prognostic, Predictive, cancer, Inflammation

## Abstract

**Background:**

Less than half of unselected metastatic cancer patients benefit from the immune checkpoint inhibitor (ICI) therapy. Systemic cancer-related inflammation may influence the efficacy of ICIs and thus, systemic inflammatory markers could have prognostic and/or predictive potential in ICI therapy. Here, we aimed to identify a combination of inflammation-related laboratory parameters to establish a practical prognostic risk model for the pretreatment evaluation of a response and survival of ICI-treated patients with different types of metastatic cancers.

**Methods:**

The study-cohort consisted of a real-world patient population receiving ICIs for metastatic cancers of different origins (n = 158). Laboratory parameters determined before the initiation of the ICI treatment were retrospectively collected. Six inflammation-related parameters i.e., elevated values of neutrophils, platelets, C-reactive protein (CRP), erythrocyte sedimentation rate (ESR) and lactate dehydrogenase (LDH), and the presence of anemia, were each scored with one point, giving 0–6 risk points for each patient. The patients with information of all these six parameters (n = 109) were then stratified into low-risk (0–3 points) and high-risk (4–6 points) groups. The overall response rate (ORR), overall survival (OS), and progression-free survival (PFS) according to the risk scores were determined.

**Results:**

The risk model was strongly associated with the outcome of the patients. The ORR to ICI treatment in the high-risk group was 30.3% in comparison to 53.9% in the low-risk group (p = 0.023). The medians for OS were 10.0 months and 27.3 months, respectively (p < 0.001), and the corresponding medians for PFS were 3.9 months and 6.3 months (p = 0.002). The risk group remained as a significant prognostic factor for both OS (HR 3.04, 95% CI 1.64–5.64, p < 0.001) and PFS (HR 1.79, 95% CI 1.04–3.06, p = 0.035) in the Cox multivariate analyses.

**Conclusions:**

We propose a readily feasible, practical risk model consisted of six inflammation-related laboratory parameters as a tool for outcome prediction in metastatic cancer patients treated with ICIs. The risk model was strongly associated with the outcome of the patients in terms of all the evaluated indicators i.e., ORR, OS and PFS. Yet, further studies are needed to validate the risk model.

**Supplementary Information:**

The online version contains supplementary material available at 10.1186/s12885-023-11699-0.

## Background

The search for biomarkers predictive of the response to immune checkpoint inhibitors (ICIs), including programmed death-1 (PD-1), programmed death ligand-1 (PD-L1) and cytotoxic T-lymphocyte-associated protein 4 (CTLA-4) inhibitors, has attracted significant attention in recent years. This is not surprising considering that less than every second unselected cancer patient benefits from the ICI therapy [1, 2]. Few tumor-derived markers, such as the expression of PD-L1 in the tumor and tumor-infiltrating immune cells, have been acknowledged as predictive and/or prognostic factors in selected tumor types, along with microsatellite instability (MSI) status and tumor mutational burden (TMB) in more diverse tumor types [3–5]. However, there are problems concerning tumor-derived biomarkers such as the technical challenges encountered in obtaining tumor material for biomarker staining. Moreover, the use of archived biopsy material can be unreliable due to the dynamic nature of the tumor microenvironment [6]. Specific issues regarding the use of PD-L1 expression as a biomarker arise from the variability in the antibodies applied in immunohistochemistry staining and different cut-off points for PD-L1 expression [3]. For these reasons, easily accessible parameters derived from blood samples would represent an attractive alternative to tissue-derived biomarkers.

Peripheral blood panels that are collected from ICI-treated patients for safety monitoring before and during the therapy comprise a potential supply for secondary use as prognostic and/or predictive biomarkers. Many of these parameters, such as the complete blood count, C-reactive protein (CRP), erythrocyte sedimentation rate (ESR) and lactate dehydrogenase (LDH) are indicators of systemic inflammation. Indeed, unlike tumor-derived biomarkers, peripheral blood inflammatory parameters may also represent the immunological state of the host in a more comprehensive manner. Although the exact mechanisms of the immunological tumor-host crosstalk are largely unknown, systemic inflammation has been acknowledged as a poor prognostic indicator in patients with cancer [7]. Furthermore, there are reports describing associations between e.g., high values of neutrophils, CRP and LDH and poor outcome among cancer patients receiving ICIs [8–10]. Considering the complexity of cancer-related inflammation and the potential overlap with other inflammatory processes, measuring a single parameter might well be a subject to error, while a multi-parameter tool could be a better option for acquiring a more reliable prediction of the response to ICIs.

Here, we propose a simple prognostic risk model for ICI-treated metastatic cancer patients in which six peripheral blood inflammation-related parameters i.e., elevated counts of neutrophils and platelets, elevated levels of CRP, ESR and LDH and the presence of anemia were each scored with one point giving 0–6 risk points for each patient. We hypothesized that the outcome of the patients with higher risk scores would be inferior to those with lower risk scores. The study cohort consisted of a real-world patient population receiving ICI therapy for metastatic cancers of different origins.

## Methods

### Patients

In this study, data from all the patients with metastatic cancers treated with PD-1/PD-L1 inhibitors in Kuopio University Hospital, Cancer Center, between January 1st, 2015, and December 31st, 2021, were retrospectively collected. In addition to patients receiving single-PD-1/-PD-L1 inhibitors, also patients receiving the combination of a PD-1 and a CTLA-4 inhibitor and those receiving PD-1/PD-L1-inhibitors in combination with chemotherapy were included in the analysis. Instead, patients that had received PD-1/PD-L1 inhibitors in clinical trials for cancer types without approved indications were excluded. The basic demographics, the sites of metastases (lymph nodes, lung, bone, liver and brain), laboratory, imaging and survival data were manually searched from the electronic patient registry of Kuopio University Hospital with the permission of the Medical Research Ethics Committee of Wellbeing Services County of North Savo (permission number 1482/2019) and organization-level permission from Kuopio University Hospital (research permit 5,654,210, 916/2022). The study was conducted in accordance with the Declaration of Helsinki; due to its retrospective nature, informed consent was not required. The methodology was guided by the REMARK criteria when applicable [11].

### Laboratory parameters and scoring method for the risk model

Peripheral blood parameters drawn 1 to 14 days before the initiation of the ICI treatment were retrospectively collected from the patients’ records. The parameters for the risk model were selected based on the following criteria: (1) the parameters were biologically related to cancer inflammation, (2) the parameters were associated with the outcome in univariate survival analyses and (3) the parameters had recognized reference ranges. With respect to the white blood cells counts (total leukocytes, neutrophils and lymphocytes), the neutrophil count was selected for the risk model as it showed the greatest significance in the univariate survival analyses (data not shown). Based on these criteria, the following six laboratory parameters were included in the risk model: hemoglobin, platelet count, neutrophil count, CRP, ESR and LDH levels. For determining the risk score cut-offs, anemia was defined as a hemoglobin level < 120 g/L based on the recommended hemoglobin target range in the treatment of cancer-related anemia [12, 13]. Elevated counts of platelets and neutrophils, and elevated levels of CRP, ESR and LDH were defined based on deviations from the normal range of variance (Eastern Finland Laboratory Centre, Kuopio, Finland) as shown in Table 1. Each of these deviations was then scored with one point, giving a minimum of zero and maximum of six points for each patient. The patients were subsequently divided into low-risk (0–3 points) and high-risk (4–6 points) groups based on their scores.

**Table 1:** Peripheral blood parameters and scoring method for the risk model

Laboratory	Risk score	Risk score
parameter	0 point	1 point
**Hemoglobin** (g/L)	≥ 120	< 120
**Platelet count** (E9/L)	≤ 360	> 360
**Neutrophil count** (E9/L)	≤ 7.5	> 7.5
**CRP** (mg/L)	≤ 3	> 3
**ESR** (mm/h)	≤ 20	> 20
**LDH** (U/L)	≤ 205	> 205

CRP, C-reactive protein; ESR, erythrocyte sedimentation rate;

LDH, lactate dehydrogenase

### Response evaluation

The response to the ICI treatment was assessed using radiologists’ RECIST-criteria based [14] evaluations of the patients’ contrast-enhanced computed tomography (CT) scans which tended to be scheduled approximately at 8 to 12 weeks intervals according to the routine clinical practice, and/or the patient’s clinical condition as assessed by the treating physicians. The information was retrospectively collected from the patient records. The best response to ICI treatment was categorized as follows; a complete response (CR), partial response (PR), stable disease (SD), progressive disease (PD) or not evaluable. The overall response rate (ORR) was defined as the percentage of the patients with CR or PR, and the disease control rate (DCR) as the percentage of the patients with CR, PR or SD [15].

The overall survival (OS) was calculated from the date of the ICI treatment initiation to the date of death from any reason or the date of the last follow-up visit, and death was counted as an event. The progression-free survival (PFS) was calculated from the date of ICI initiation to a progression of the cancer, death from any cause or the end of the follow-up, whichever occurred first, and tumor progression or death were counted as events. The numbers of OS and PFS events in the whole patient cohort and in the investigated subgroups are shown in Additional file 1.

### Statistical analyses

All the data were analyzed with SPSS statistics software (Armonk, NY, USA), version 27. Differences between the groups were analyzed with the Chi-square or the Fisher’s Exact tests. The survival curves were plotted with the Kaplan-Meier method and the groups were compared with the log rank test. Univariate and multivariate survival analyses were performed with the Cox’s model. The median follow-up time was determined using the reverse Kaplan-Meier method. A probability value of less than 0.05 was considered statistically significant.

## Results

### Demographics of the patients

There were 158 metastatic cancer patients treated with ICIs between 2015 and 2021. The basic characteristics of the patients at the time of ICI initiation are presented in Table 2. Information about the hemoglobin level and platelet count were available for all the patients, neutrophil count for 157 (99.4%), CRP for 124 (78.5%), ESR for 111 (70.3%) and LDH for 119 (75.3%) patients. Altogether, 109 (69.0%) patients had information available for all of the six parameters included in the risk model. The number of risk points distributed as follows: 0 points n = 7 (6.4%), 1 point n = 19 (17.4%), 2 points n = 21 (19.3%), 3 points n = 29 (26.6%), 4 points n = 18 (16.5%), 5 points n = 12 (11.0%) and 6 points n = 3 (2.8%). The risk score was 0–3 in 69.7% (n = 76) of the patients (referred to as the low-risk group) and 4–6 in 30.3% (n = 33) of the patients (referred to as the high-risk group).

**Table 2:** Basic demographics of the patients

	All	Risk model n = 109 n (%)	Low-risk n = 76 n (%)	High-risk n = 33 n (%)	P value
	n = 158				Low vs.
	n (%)				high risk
**Sex**					0.302
Male	108 (68.4%)	75 (68.8%)	50 (65.8%)	25 (75.8%)	
Female	50 (31.6%)	34 (31.2%)	26 (34.2%)	8 (24.2%)	
**Age**					0.923
≤ 65 years	76 (48.1%)	47 (43.1%)	33 (43.4%)	14 (42.4%)	
> 65 years	82 (51.9%)	62 (56.9%)	43 (56.6%)	19 (57.6%)	
**PS (WHO)**					0.003*
0	92 (58.2%)	66 (60.6%)	53 (69.7%)	13 (39.4%)	
≥ 1	66 (41.8%)	43 (39.4%)	23 (30.3%)	20 (60.6%)	
**Cancer type**					0.288
NSCLC	75 (47.5%)	52 (47.7%)	37 (48.7%)	15 (45.5%)	
Melanoma	28 (17.7%)	19 (17.4%)	16 (21.1%)	3 (9.1%)	
RCC	26 (16.5%)	19 (17.4%)	10 (13.2%)	9 (27.3%)	
Head and neck	8 (5.1%)	7 (6.4%)	3 (3.9%)	4 (12.1%)	
MSI high	7 (4.4%)	5 (4.6%)	4 (5.3%)	1 (3.0%)	
Urothelial	8 (5.1%)	4 (3.7%)	3 (3.9%)	1 (3.0%)	
Lymphoma	5 (3.2%)	2 (1.8%)	2 (2.6%)	0 (0%)	
TNBC	1 (0.6%)	1 (0.9%)	1 (1.3%)	0 (0%)	
**Metastatic sites**					
Lymph nodes	104 (65.8%)	71 (65.1%)	46 (60.5%)	25 (75.8%)	0.125
Lung	89 (56.3%)	60 (55.0%)	44 (57.9%)	16 (48.5%)	0.364
Bone	47 (29.7%)	33 (30.3%)	17 (22.4%)	16 (48.5%)	0.006*
Liver	33 (20.9%)	27 (24.8%)	16 (21.1%)	11 (33.3%)	0.172
Brain	17 (10.8%)	12 (11.0%)	8 (10.5%)	4 (12.1%)	0.752

PS, performance status; WHO, World Health Organization; NSCLC, non-small cell lung cancer; RCC, renal cell carcinoma; MSI, microsatellite instability; TNBC, triple negative breast cancer; *p < 0.05

Of the 109 patients included in the risk model, 68.8% (n = 75) were male. The median age of the patients at the time of ICI initiation was 67.9 years (range 33.5–87.2). The median follow-up time was 23.9 months, (95% CI 18.9–28.9). The majority of the patients had a good performance status (PS) i.e., 0–1 (96.3%, n = 105). Patients in the high-risk group had more frequently PS ≥ 1 as compared to the low-risk group, i.e., 60.6% vs. 30.3%, respectively (p = 0.003). The most common cancer type was non-small cell lung cancer (NSCLC, 47.7%, n = 52), followed by melanoma (17.4%, n = 19) and renal cell carcinoma (RCC, 17.4%, n = 19). The occurrence of cancer metastases was assessed in the following sites: lymph nodes, lung, bone, liver and brain with lymph nodes (65.1%, n = 71) and lung (55.0%, n = 60) being the most common sites of metastases. Bone metastases were more frequent among the patients in the high-risk group in comparison to the low-risk group, i.e., 48.5% vs. 22.4%, respectively (p = 0.006). Otherwise, there were no statistically significant differences in the basic demographics between the low and high-risk groups. (Table 2)

The ICI treatments received by the patients are presented in detail in Additional file 2. Of the patients included in the risk model, 61.5% (n = 67) received an ICI as the first line treatment for their metastatic disease. The majority of the patients (67.9%, n = 74) received a single PD-1/PD-L1 inhibitor, 23.9% (n = 26) were administered a combination of PD-1/PD-L1 inhibitor with chemotherapy and 8.3% (n = 9) were treated with a combination of PD-1 inhibitor and CTLA-4 inhibitor (i.e., nivolumab and ipilimumab). The treatments distributed similarly in the low and high-risk groups (Additional file 2).

### Responses to ICI treatment according to the risk scores

Responses to ICI treatment according to the risk groups are shown in Table 3. Both ORR and DCR to ICI treatment were inferior in the high-risk group as compared to the low-risk group. The ORR was 30.3% (10/33) in the high-risk group as compared to 53.9% (41/76) in the low-risk group (p = 0.023). Similarly, the DCR was 42.4% (14/33) in the high-risk group and 75.0% (57/76) in the low-risk group (p = 0.001). For one patient in the high-risk group the response was not evaluable as the patient had died before any response assessment was performed. When the ORR was evaluated against each risk score i.e., from zero to six, the ORR gradually decreased as the risk score increased (Table 4). The ORR was the highest, 85.7%, among the patients with zero risk points (n = 7) whereas none of the patients with six risk points (n = 3) displayed a response.

**Table 3:** Responses to ICI treatment according to the risk groups

Best response to ICI	All	Low-risk n = 76 n (%)	High-risk n = 33 n (%)	P value
	n = 109			Low vs.
	n (%)			high risk
**Complete response**	10 (9.2%)	9 (11.8%)	1 (3.0%)	
**Partial response**	41 (37.6%)	32 (42.1%)	9 (27.3%)	
**Stable disease**	20 (18.3%)	16 (21.1%)	4 (12.1%)	
**Progressive disease**	37 (33.9%)	19 (25.0%)	18 (54.5%)	
**Not evaluable**	1 (0.9%)	0 (0%)	1 (3.0%)	0.012*

ICI, immune checkpoint inhibitor: *p < 0.05

**Table 4:** Responses and survival according to the risk scores

Number of	Patients	ORR	mOS (95% CI)	mPFS (95% CI)
risk points	n (%)	%	months	months
**0**	7 (6.4%)	85.7%	not reached	not reached
**1**	19 (17.4%)	52.6%	22.2 (12.9–31.6)	6.2 (1.9–10.6)
**2**	21 (19.3%)	52.4%	29.7 (9.0-50.3)	6.6 (3.3–9.9)
**3**	29 (26.6%)	48.3%	20.9 (N/A)	5.2 (3.2–7.3)
**4**	18 (16.5%)	44.4%	10.0 (7.5–12.6)	4.2 (3.2–5.3)
**5**	12 (11.0%)	16.7%	8.4 (0.0-19.7)	2.0 (1.4–2.7)
**6**	3 (2.8%)	0%	2.0 (0.0-5.1)	1.6 (0.0-4.2)

ORR, overall response rate; mOS, median overall survival; CI, confidence interval; mPFS, median progression-free survival; N/A, not available

### Survival according to the risk scores

**Fig. 1:**
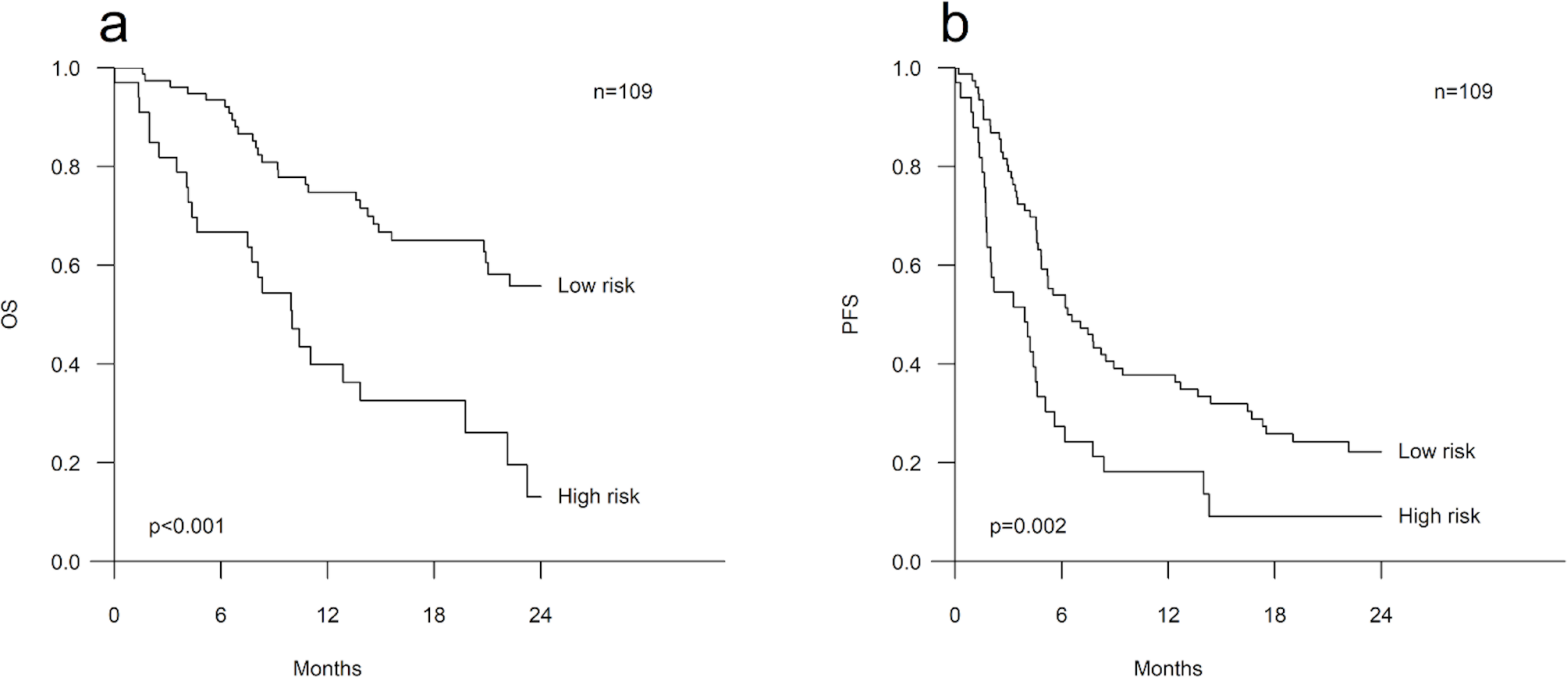
Survival of the patients according to the risk groups. Kaplan-Meier curves illustrating overall survival (OS) (**a**) and progression-free survival (PFS) (**b**) according to the risk groups among all the patients included in the risk model

During the follow-up, 58 (53.2%) of the patients included in the risk model had died. The OS of the patients in the high-risk group was significantly worse in comparison to the patients in the low-risk group (HR 3.22, 95% CI 1.87–5.55, p < 0.001). The median OS was 10.0 months (95% CI 7.1–12.9) in the high-risk group whereas it was significantly longer, 27.3 months (95% CI 19.6–35.0), in the low-risk group (p < 0.001) (Fig. 1a). PFS was also inferior in the high-risk group as compared to the low-risk group (HR 2.03, 95% CI 1.29–3.21, p = 0.002), and the medians for PFS were 3.9 months (95% CI 1.4–6.4) and 6.3 months (95% CI 3.7-9.0), respectively (p = 0.002) (Fig. 1b). The high-risk group remained as a significant prognostic factor for both poor OS (HR 3.04, 95% CI 1.64–5.64, p < 0.001) and PFS (HR 1.79, 95% CI 1.04–3.06, p = 0.035) in the Cox multivariate survival analyses including several clinical parameters i.e., sex, age, PS, the line of therapy and metastatic sites (Table 5). Furthermore, the high-risk group was associated with inferior OS and PFS regardless of the type of ICI treatment, i.e., among the patients treated with only ICI (a single PD-1/PD-L1 inhibitor or the combination of nivolumab and ipilimumab, n = 83) as well as among the patients treated with a combination of a PD-1/PD-L1 inhibitor and chemotherapy (n = 26) (p ≤ 0.041) (Additional file 3).

**Table 5:** COX multivariate analyses for OS and PFS

OS	HR	95% CI	P value	PFS	HR	95% CI	P value
**High-risk group**	3.04	1.64–5.64	< 0.001*	**High-risk group**	1.79	1.04–3.06	0.035*
**Male sex**	1.55	0.83–2.90	0.172	**Male sex**	0.89	0.55–1.46	0.654
**Age > 65 years**	0.72	0.42–1.24	0.238	**Age > 65 years**	0.77	0.49–1.19	0.240
**PS (WHO) ≥ 1**	1.30	0.68–2.50	0.428	**PS (WHO) ≥ 1**	1.28	0.75–2.17	0.366
**Therapy line > 1**	1.12	0.63–1.98	0.706	**Therapy line > 1**	0.98	0.59–1.62	0.939
**Nodal metastases**	0.93	0.53–1.63	0.789	**Nodal metastases**	0.89	0.56–1.40	0.608
**Lung metastases**	0.99	0.57–1.71	0.960	**Lung metastases**	1.37	0.87–2.16	0.169
**Bone metastases**	0.91	0.50–1.65	0.751	**Bone metastases**	1.76	1.05–2.94	0.032*
**Liver metastases**	2.80	1.55–5.07	0.001*	**Liver metastases**	1.90	1.12–3.20	0.017*
**Brain metastases**	2.28	0.97–5.35	0.060	**Brain metastases**	2.26	1.16–4.43	0.017*

OS, overall survival; PFS, progression-free survival; HR, hazard ratio; CI, confidence interval; PS, performance status; WHO, World Health Organization; * p < 0.05.

**Fig. 2:**
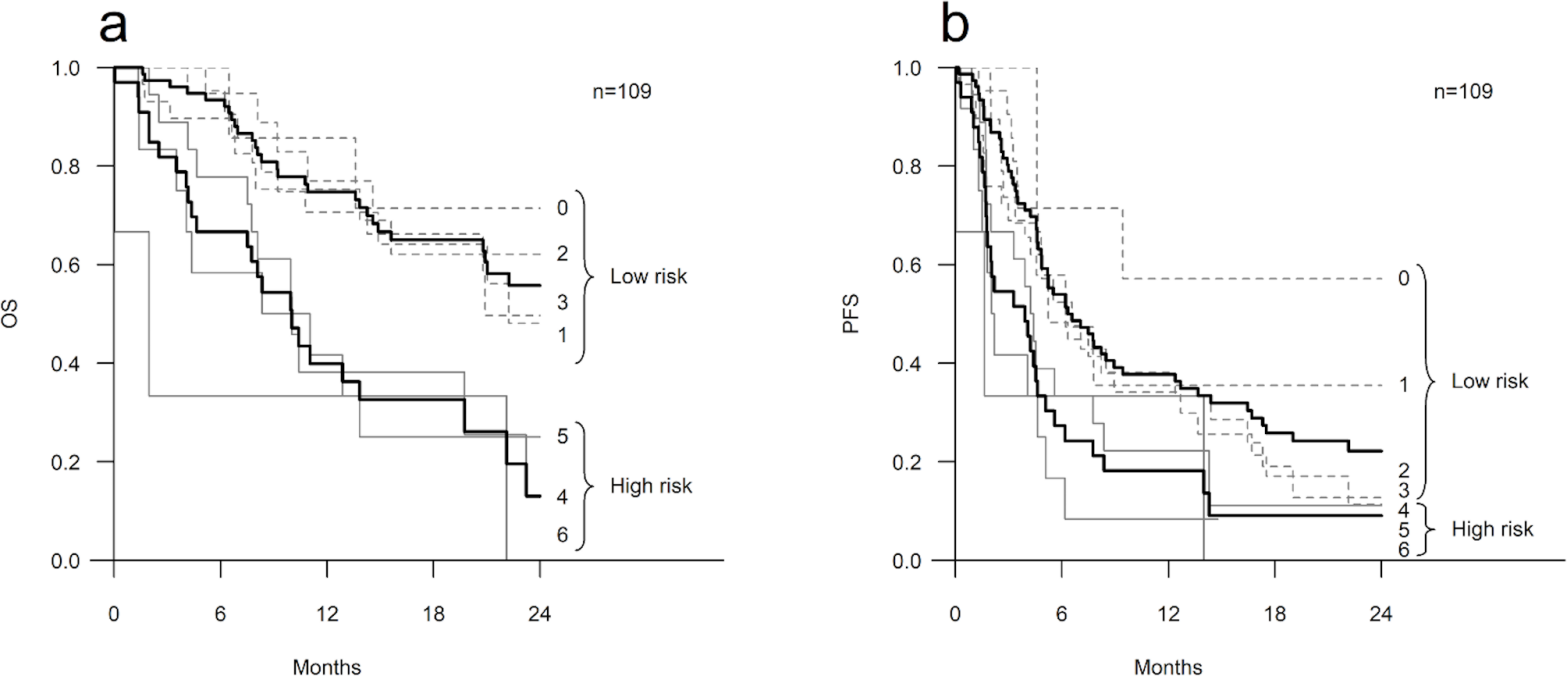
Survival of the patients according to the risk scores. Kaplan-Meier curves illustrating overall survival (OS) (**a**) and progression-free survival (PFS) (**b**) according to the risk scores from zero to six. Both OS and PFS gradually decreased as the risk score increased. Thick black lines represent the risk groups

When the survival was evaluated against each risk score from zero to six, both OS and PFS gradually decreased as the risk score increased (Table 4; Fig. 2). For the patients with zero risk points (n = 7) the medians for OS and PFS were not reached. In contrast, among the patients with five or six risk points (n = 12 and n = 3, respectively), the medians for OS and PFS were the shortest, 8.4 and 2.0 months and 2.0 and 1.6 months, respectively (Table 4).

All the laboratory parameters included in the risk model were associated with OS and/or PFS also when investigated separately (Additional file 4). Of the clinical parameters, sex, PS, the line of therapy and the presence of bone, liver or brain metastases were significantly associated with OS and/or PFS (Additional file 4).

### Survival analyses stratified by the Tumor type

**Fig. 3:**
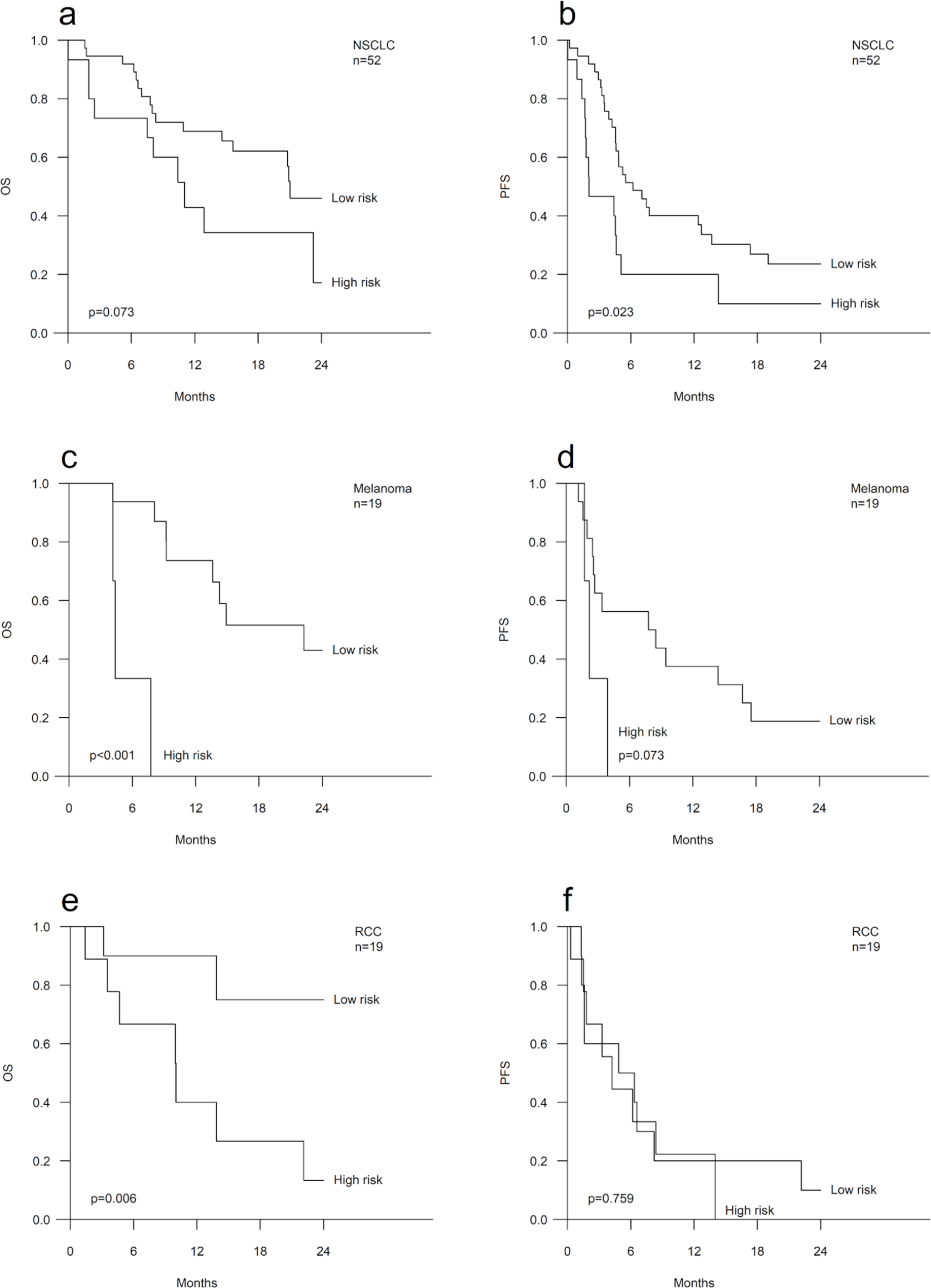
Survival of the patients in the largest histological subgroups. Kaplan-Meier curves showing overall survival (OS) and progression-free survival (PFS) according to the risk groups among the patients with non-small cell lung cancer (NSCLC) (**a, b**), melanoma (**c, d**) and renal cell carcinoma (RCC) (**e, f**)

The most common tumor type was NSCLC (n = 52), in which the high-risk group was associated with inferior PFS (HR 2.13, 95% CI 1.09–4.16, p = 0.026) and a similar but a non-significant trend was detected for OS (HR 2.04, 95% CI 0.92–4.53, p = 0.079) (Fig. 3a-b, Additional file 3). In addition, among the patients with melanoma (n = 19), being in the high-risk group was associated significantly with inferior OS (HR 22.72, 95% CI 2.27-227.26, p = 0.008) and this was also the case for those with renal cell carcinoma (n = 19, HR 6.71, 95% CI 1.39–32.32, p = 0.018) (Fig. 3c-f, Additional file 3). The other histological subgroups were not evaluated separately since there were even smaller numbers of patients.

## Discussion

In this study with a real-world population of 158 ICI-treated metastatic cancer patients, a practical peripheral blood-based risk model for survival prediction was developed. In the risk model, six inflammation-related parameters i.e., the presence of anemia, elevated counts of neutrophils and platelets and elevated levels of CRP, ESR and LDH, were each awarded one point and combined, i.e., a score of 0–6 risk points for each patient. The information of all these six laboratory parameters determined before the initiation of the ICI therapy was available for 109 patients, and based on the risk scores, the patients were stratified into either low-risk (0–3 points) or high-risk (4–6 points) groups. We found that OS, PFS and response rates were all inferior among the patients in the high-risk group as compared to the patients in the low-risk group. Importantly, the risk group remained as a significant prognostic factor for both OS and PFS in the Cox multivariate analyses even when including several clinical parameters, suggesting that it does have an independent role in predicting the outcome of metastatic cancer patients treated with ICIs.

The risk model was strongly associated with the outcome of the patients in terms of all the evaluated indicators of outcome i.e., OS, PFS and response rate. In the high-risk group, median OS was only 10.0 months as compared to 27.3 months in the low-risk group (p < 0.001), and the corresponding medians for PFS were 3.9 months and 6.3 months (p = 0.002). Even though there were more patients with poorer PS (PS ≥ 1) in the high-risk group, the risk group remained as a significant prognostic factor for both OS (HR 3.04, 95% CI 1.64–5.64, p < 0.001) and PFS (HR 1.79, 95% CI 1.04–3.06, p = 0.035) in the Cox multivariate analyses when supplemented with PS and other clinical variables, highlighting its independent role of PS in predicting the outcome. In addition to the prognostic significance, the risk model seemed to have predictive value since the ORR to ICI treatment was only 30.3% in the high-risk group as compared to 53.9% in the low-risk group (p = 0.023). Furthermore, as the risk points increased from zero to six, there was evidence of a progressive worsening of the patients’ outcomes (Table 4).

Previously, various prognostic nomograms have been suggested for ICI therapy with different tumor types. For example, Mezquita et al. have reported the association of a high LIPI index, i.e., an index based on pretreatment levels of LDH and derived neutrophil-to-lymphocyte ratio (dNLR), with worse outcomes of ICI therapy in NSCLC patients [16] and Pan et al. have proposed a combinatory index of dNLR and platelet-to-lymphocyte ratio (PLR) as a prognostic factor in gastric cancer patients receiving ICI therapy [17]. As far as we are aware, the risk model presented here is the first nomogram constituted from a histology-agnostic perspective for the outcome prediction of ICI-treated cancer patients. Our real-world patient cohort included metastatic cancer patients with various tumor types. The risk model appeared to be functional in all the major subgroups stratified by the tumor type i.e., NSCLC, melanoma and renal cell carcinoma (Fig. 3, Additional file 3). The strongest prognostic contribution appeared among melanoma patients, but that finding remains somewhat uncertain due to the low number of patients in the subgroup analyses. However, in this highly immunogenic tumor type [18], strong associations between circulating inflammatory factors and outcomes of ICI treatment have been previously reported [9, 10, 19, 20]. Furthermore, our risk model was associated with survival regardless of the ICI treatment type, i.e., among the patients treated with ICI only as well as among the patients treated with a PD-1/PD-L1 inhibitor and chemotherapy, which is important as ICIs are increasingly administered in combination with other agents.

Some publications have also incorporated clinical risk factors, such as PS, age and the presence of liver metastases, in their prognostic/predictive nomograms [21, 22]. Considering our inflammation-related focus, clinical factors were not incorporated into the risk model of the current study. However, associations between the risk groups and several clinical variables, such as PS, the sites of metastases, the type of ICI treatment and the line of therapy were evaluated. We observed that the patients in the high-risk group had more often a poorer PS (≥ 1) and a higher frequency of bone metastases; this finding may reflect their higher metastatic burden, which is in line with previous data showing that cancer-related inflammation can promote the metastatic capacity of a tumor [23].

The decision of including whole blood parameters (platelet count, neutrophil count and hemoglobin) and soluble inflammatory mediators (CRP, ESR and LDH) in our risk model was based on their easy accessibility in clinical routine. From a biological viewpoint, these parameters reflect the systemic cancer promoting inflammation acting via different underlying mechanisms [24]. Neutrophils mediate tumor progression and resistance to cancer therapies e.g., by suppressing the functions of tumor-infiltrating lymphocytes and dendritic cells [25]. A high NLR, i.e., a relative increase in the numbers of neutrophils accompanied by a decrease in those of lymphocytes in the blood, has been associated with a poor outcome in several cancers and there is also evidence of a high NLR associating with inferior survival among ICI-treated cancer patients [26–28]. Platelets contribute to the proliferation of cancer cells and metastatic and angiogenic capacity via multiple routes, such as transforming growth factor beta (TGF-β) and vascular endothelial growth factor (VEGF) -integrin signaling [29]. An elevated platelet count on its own or in relation to the blood lymphocyte count (PLR) has been shown to predict a poor survival of cancer patients [30–32]. CRP is a rapidly appearing marker of tissue damage and persistently elevated levels of CRP induced by inflammatory cytokines, correlate with chronic tissue irritation such as cancer-related inflammation [33]. Several investigators have reported associations between an elevated CRP level and poor survival in ICI-treated cancer patients [9, 34]. ESR reflects the inflammatory status of a patient, and it may be elevated in any inflammatory condition inducing fibrinogen production and anemia [35]. An elevated ESR value has been proposed to be a predictor of poor survival in patients with cancer [36, 37]. The aerobic glycolysis which is present in cancer cells is enzymatically regulated by LDH [38], and a high serum LDH level has been strongly associated with tissue destruction, tumor cell immune evasion and modest outcomes in a variety of cancers, such as melanoma [39] and lymphomas, where it has been integrated into the prognostic scoring system and plays a role in the therapeutic decision-making protocols [40]. Anemia due to a chronic disease, such as cancer, results from the dysregulation of iron homeostasis induced by inflammatory cytokines such as interleukin-6 (IL-6) and interferon gamma (IFN-γ) as well as the suppression of erythropoietic activity, and it associates with poor OS among cancer patients [41–43].

In line with this biological background, all the six inflammation-related parameters included in our risk model were associated with significantly weaker OS and/or PFS also when investigated separately. It is important to point out, however, that none of these parameters are specific to cancer-related inflammation and may be altered due to several confounding factors, e.g., neutrophilia due to microbial infections or anemia due to bleeding, which may contribute to poorer survival independently of the presence of a cancer. The patients with possible confounding factors were not excluded from the present study due to challenges in recognizing and categorizing these diverse and sometimes chronic conditions. To increase methodological specificity, high-content data-generating methods such as the analysis of peripheral immune-cell subtypes by flow/mass cytometry or transcriptomic profiling should be employed. Previous flow cytometry studies have indeed been able to link the specific baseline leukocyte subtypes and their longitudinal changes during ICI therapy with responsiveness and prognosis of cancer patients treated with ICIs. For example, an increase in the number of circulating cytotoxic (Ki67 + CD8 + PD-1+) lymphocytes [44], the ratio of central memory to effector T lymphocytes subtype [45] and the higher frequency of blood NK cells [46] and classical monocytes (CD14 + CD16 + HLA-DR+) at baseline [47] appear to correlate with improved survival in PD-1/PD-L1inhibitor treated lung cancer and melanoma patients. Interestingly, it also appears that multicolor flow cytometry-based peripheral immune-cell phenotyping can also be functional in a tumor-agnostic setting. In a recent study, Zhou et al. were able to provide a liquid immune-profile-based signature to predict the likelihood of the survival of ICI-treated cancer patients with variable tumor histologies [48]. Nevertheless, as the immunological mechanisms influencing cancer progression and ICI responses are complex, it seems inevitable that a prognostic model combining several immune-associated parameters is an efficient way to improve specificity in contrast to a single-biomarker approach. Further research, however, is needed to uncover, validate and standardize the most powerful yet cost-effective combination of biomarkers for accurate and clinically meaningful sensitivity, specificity and reproducibility.

In addition to its prognostic significance, our risk model seemed to have predictive value since the higher risk scores were associated with lower response rates to ICI treatment (Tables 3 and 4). As ICI therapy is based on restoring anti-tumor immunity, it is reasonable to postulate that the systemic inflammatory parameters in the therapy-naïve state could serve as markers of the individual’s subsequent sensitivity to ICIs. Indeed, mass cytometry analyses of peripheral blood immune cells have already been able to reveal specific compositions of immune cells which are predictive of the likelihood of a response to ICIs [47, 49, 50]. Importantly, the study of Mezquita et al. included a chemotherapy-treated control cohort in which the association of the LIPI index with survival could not be significantly demonstrated, suggesting the predictive value of LDH and dNLR especially in the ICI-treated NSCLC patient population [16]. In the present study, due to the lack of a control patient cohort with an alternative treatment, it remains to be confirmed whether the risk model is predictive of the ICI treatment result or whether it is prognostic of the disease outcome in general. Furthermore, as the results are based on retrospective single-center data without a validation cohort, further studies are needed to validate the risk model.

## Conclusions

Prognostic and predictive markers for cancer patients treated with ICIs are needed in the clinical practice due to the costs and immune-related toxicities of this form of treatment. Here, we devised a practical risk model based on six inflammation-related routine blood tests as a tool for outcome prediction in metastatic cancer patients being treated with ICIs. We found that in terms of all the evaluated indicators of outcome i.e., OS, PFS and response rate, the risk model was strongly associated with the outcome of the patients. Since the risk model was based on routine blood tests, it is both feasible and inexpensive and thus could easily be incorporated into the clinical practice. Further studies examining larger patient populations are needed to validate the risk model.

### Electronic supplementary material

Below is the link to the electronic supplementary material.

Supplementary Material 1

## Data Availability

The datasets used and/or analyzed during the current study are available from the corresponding author on reasonable request.

## Abbreviations

CI: Confidence interval
CR: Complete response
CRP: C-reactive protein
CTLA-4: Cytotoxic T-lymphocyte-associated protein 4
DCR: Disease control rate
dNLR: Derived neutrophil-to-lymphocyte ratio
ESR: Erythrocyte sedimentation rate
HR: Hazard ratio
ICI: Immune checkpoint inhibitor
IFN-γ: Interferon gamma
IL-6: Interleukin-6
LDH: Lactate dehydrogenase
MSI: Microsatellite instability
NLR: Neutrophil-to-lymphocyte ratio
NSCLC: Non-small cell lung cancer
ORR: Overall response rate
OS: Overall survival
PD: Progressive disease
PD-1: Programmed death-1
PD-L1: Programmed death ligand-1
PFS: Progression-free survival
PLR: Platelet-to-lymphocyte ratio
PR: Partial response
PS: Performance status
RCC: Renal cell carcinoma
SD: Stable disease
TGF-β: Transforming growth factor beta
TMB: Tumor mutational burden
VEGF: Vascular endothelial growth factor
