# Supplementary material for: A practical prognostic peripheral blood-based risk model for the evaluation of the likelihood of a response and survival of metastatic cancer patients treated with immune checkpoint inhibitors
Source: BMC Cancer. 2023 Dec 4;23:1186. doi: 10.1186/s12885-023-11699-0 (PMC10694914; doi:10.1186/s12885-023-11699-0)
Supplement: Supplementary file 1 — Supplementary Material 1 [file 12885_2023_11699_MOESM1_ESM.pdf]

Additional file 1 The numbers of OS and PFS events in the investigated patient groups

	**Number of patients**	**OS event* n (%)**	**PFS event** n (%)**
**All**	158	88 (55.7 %)	130 (82.3 %)
**Risk model**	109	58 (53.2 %)	87 (79.8 %)
High-risk group	33	25 (75.8 %)	29 (87.9 %)
Low-risk group	76	33 (43.4 %)	58 (76.3 %)
**ICI only**	83	48 (57.8 %)	69 (83.1 %)
High-risk group	27	21 (77.8 %)	23 (85.2 %)
Low-risk group	56	27 (48.2 %)	46 (82.1 %)
**ICI and chemotherapy**	26	10 (38.5 %)	18 (69.2 %)
High-risk group	6	4 (66.7 %)	6 (100 %)
Low-risk group	20	6 (30.0 %)	12 (60.0 %)
**NSCLC**	52	28 (53.8 %)	40 (76.9 %)
High-risk group	15	10 (66.7 %)	13 (86.7 %)
Low-risk group	37	18 (48.6 %)	27 (73.0 %)
**Melanoma**	19	12 (63.2 %)	17 (89.5 %)
High-risk group	3	3 (100 %)	3 (100 %)
Low-risk group	16	9 (56.3 %)	14 (87.5 %)
**Renal cell carcinoma**	19	10 (52.6 %)	17 (89.5 %)
High-risk group	9	8 (88.9 %)	8 (88.9 %)
Low-risk group	10	2 (20.0 %)	9 (90.0 %)

OS, overall survival; PFS, progression-free survival; ICI, immune checkpoint inhibitor; NSCLC, non-small cell lung cancer; *OS event=death; **PFS event= tumor progression or death

Additional file 2 ICI treatments of the patients

	**All**<br>n=158<br>n (%)	**Risk model**<br>n=109<br>n (%)	**Low-risk**<br>n=76<br>n (%)	**High-risk**<br>n=33<br>n (%)	**P value**<br>Low vs.<br>high risk
**Line of ICI therapy**					0.395
1	84 (53.2 %)	67 (61.5 %)	49 (64.5 %)	18 (54.5 %)	
2	49 (31.0 %)	28 (25.7 %)	16 (21.1 %)	12 (36.4 %)	
3	14 (8.9 %)	7 (6.4 %)	5 (6.6 %)	2 (6.1 %)	
≥4	11 (7.0 %)	7 (6.4 %)	6 (7.9 %)	1 (3.0 %)	
**PD-(L)1 inhibitor only**	**119 (75.3 %)**	**74 (67.9 %)**	**51 (67.1 %)**	**23 (69.7 %)**	0.468
Pembrolizumab	60 (38.0 %)	41 (37.6 %)	29 (38.2 %)	12 (36.4 %)	
Nivolumab	55 (34.8 %)	30 (27.5 %)	19 (25.0 %)	11 (33.3 %)	
Atezolizumab	4 (2.5 %)	3 (2.8 %)	3 (3.9 %)	0 (0 %)	
**PD-(L)1 inhibitor with chemotherapy**	**30 (19.0 %)**	**26 (23.9 %)**	**20 (26.3 %)**	**6 (18.2 %)**	
Pembrolizumab + chemotherapy	29 (18.4 %)	25 (22.9 %)	19 (25.0 %)	6 (18.2 %)	
Atezolizumab + chemotherapy	1 (0.6 %)	1 (0.9 %)	1 (1.3 %)	0 (0 %)	
**Nivolumab + ipilimumab**	**9 (5.7 %)**	**9 (8.3 %)**	**5 (6.6 %)**	**4 (12.1 %)**	

ICI, immune checkpoint inhibitor; PD-(L)1, programmed death-1/programmed death ligand-1

Additional file 3 COX univariate analyses for OS and PFS in the largest subgroups

OS	HR	95% CI	P value	PFS	HR	95% CI	P value
**ICI only (n=83)**				**ICI only (n=83)**			
High-risk group	2.98	1.63-5.44	<0.001*	High-risk group	1.70	1.02-2.84	0.041*
**ICI and chemotherapy (n=26)**				**ICI and chemotherapy (n=26)**			
High-risk group	4.32	1.13-16.45	0.032*	High-risk group	3.66	1.34-9.96	0.011*
**NSCLC (n=52)**				**NSCLC (n=52)**			
High-risk group	2.04	0.92-4.53	0.079	High-risk group	2.13	1.09-4.16	0.026*
**Melanoma (n=19)**				**Melanoma (n=19)**			
High-risk group	22.72	2.27-227.26	0.008*	High-risk group	3.27	0.83-12.89	0.090
**Renal cell carcinoma (n=19)**				**Renal cell carcinoma (n=19)**			
High-risk group	6.71	1.39-32.32	0.018*	High-risk group	1.17	0.43-3.13	0.761

OS, overall survival; PFS, progression-free survival; HR, hazard ratio; CI, confidence interval; ICI, immune checkpoint inhibitor; NSCLC, non-small cell lung cancer; * p<0.05

Additional file 4 COX univariate analyses for OS and PFS according to each laboratory parameter included in the risk model (A) and clinical parameters (B)

A. Laboratory parameters

**OS**	**HR**	**95% CI**	**P value**	**PFS**	**HR**	**95% CI**	**P value**
**Hb <120** n=158	2.15	1.37-3.38	0.001*	**Hb <120** n=158	1.44	0.97-2.14	0.072
**Platelets >360** n=158	1.71	1.10-2.66	0.016*	**Platelets >360** n=158	1.55	1.08-2.24	0.018*
**Neutrophils >7.5** n=157	2.16	1.23-3.79	0.008*	**Neutrophils >7.5** n=157	1.45	0.89-2.36	0.141
**CRP >3** n=124	2.04	1.11-3.75	0.022*	**CRP >3** n=124	2.18	1.32-3.59	0.002*
**ESR >20** n=111	2.14	1.23-3.72	0.007*	**ESR >20** n=111	1.68	1.09-2.61	0.020*
**LDH >205** n=119	1.64	0.91-2.94	0.101	**LDH >205** n=119	1.66	1.03-2.69	0.038*

B. Clinical parameters, n=158

**OS**	**HR**	**95% CI**	**P value**	**PFS**	**HR**	**95% CI**	**P value**
**Male sex**	1.65	1.02-2.66	0.041*	**Male sex**	1.43	0.97-2.10	0.072
**Age >65 years**	0.68	0.45-1.04	0.077	**Age >65 years**	0.84	0.60-1.18	0.318
**PS (WHO) ≥1**	2.29	1.49-3.51	<0.001*	**PS (WHO) ≥1**	2.05	1.44-2.92	<0.001*
**Therapy line >1**	1.56	1.02-2.38	0.040*	**Therapy line >1**	1.34	0.95-1.89	0.099
**Nodal metastases**	0.80	0.52-1.24	0.320	**Nodal metastases**	0.86	0.60-1.24	0.426
**Lung metastases**	0.88	0.58-1.35	0.564	**Lung metastases**	1.08	0.76-1.53	0.681
**Bone metastases**	1.75	1.14-2.70	0.011*	**Bone metastases**	1.92	1.32-2.79	0.001*
**Liver metastases**	2.99	1.88-4.74	<0.001*	**Liver metastases**	2.44	1.62-3.69	<0.001*
**Brain metastases**	2.57	1.39-4.76	0.003*	**Brain metastases**	2.06	1.21-3.48	0.007*

OS, overall survival; PFS, progression-free survival; HR, hazard ratio; CI, confidence interval; Hb, hemoglobin; CRP, C-reactive protein; ESR, erythrocyte sedimentation rate; LDH, lactate dehydrogenase; PS, performance status; WHO, World Health Organization; * p<0.05
